# Unravelling the mechanisms by which chronic hepatitis B infection is associated with an increased risk of gestational diabetes

**DOI:** 10.3389/fgwh.2023.1184090

**Published:** 2023-05-30

**Authors:** Subashree Paramasivam, Sushena Krishnaswamy, Michelle L Giles

**Affiliations:** ^1^ Department of Obstetrics and Gynaecology (Monash Health) Melbourne, VIC, Australia; ^2^ Department of Obstetrics and Gynaecology (Monash University), Melbourne, VIC, Australia

**Keywords:** viral hepatitis, insulin resistance, pregnancy, chronic inflammation, viral DNA load

## Abstract

An independent association between chronic hepatitis B virus (HBV) and the development of gestational diabetes (GDM) has been reported in the literature. Ethnic background and regional influences have been demonstrated to play a role in the reporting of incidence rates of GDM among women with chronic HBV. The mechanisms behind this association are poorly understood, but evidence suggests an inflammatory basis. Viral factors such as chronic HBV replication, quantifiable by HBV viral load, have been proposed to contribute to the increasing risk of insulin resistance in pregnancy. More research is needed to better characterise the association and determine if any interventions early in pregnancy for women infected with chronic HBV would mitigate the development of GDM.

## Introduction

Chronic HBV infection is a major public health challenge with a prevalence of 3.5% and 257 million people living with chronic HBV worldwide (1). Mother to child transmission (MTCT) during parturition is the most common mode of acquisition in high prevalence regions, with a 90% risk of developing chronic HBV compared to 10% following infection in adulthood (2). In addition to the risk of MTCT of HBV, recent studies have also examined associations between chronic HBV and numerous adverse pregnancy outcomes (3–21) one of which is an increased risk of developing gestational diabetes mellitus (GDM), defined as any degree of glucose intolerance first diagnosed in pregnancy (22).

GDM is associated with a number of adverse outcomes and complications in pregnancy, such as an increased risk of preterm birth, pre-eclampsia and fetal macrosomia resulting in a higher risk of shoulder dystocia and/or operative delivery (23). Neonates of pregnancies complicated by GDM are also at a higher risk of hypoglycaemia and subsequent brain injury if not appropriately managed (23). Furthermore, GDM predisposes women to an increased lifetime risk of type 2 diabetes mellitus (T2DM) and cardiovascular disease (CVD); and *via* epigenetic modifications can expose babies of mothers with GDM to an increased risk of childhood obesity, T2DM, CVD and associated metabolic diseases (23).

Several epidemiological studies have reported an association between chronic HBV and the development of GDM, even after traditional risk factors for GDM were taken into account such as body mass index (BMI), age, parity, smoking status and region of birth (3–7, 11, 12, 18–21). Different statistical approaches have been applied across the different studies, restricting direct comparisons. However even after considering these differences the magnitude of this association varies from an adjusted incidence risk ratio of 1.2 (5), relative risk 3.5 (21) and adjusted odds ratio of 1.41 (11). This paper aims to review the existing literature exploring the risk of developing GDM amongst pregnant women with chronic HBV and possible mechanisms for this association.

### Epidemiology of HBV

The epidemiological distribution of HBV can be broadly classified into high- (>8%), intermediate- (2%–7%) and low-prevalence (<2%) areas (24). Globally, it has been estimated that 45% of the world's population lives in an area of high prevalence (25), particularly in the Asia Pacific and sub-Saharan Africa. These regions are characterised by perinatal or vertical modes of transmission (2, 26), and a predominance of HBV genotypes B and C (26). People living in low-HBV-prevalence countries make up the minority of the global population (∼12%), and include Australia, Northern and Western Europe, Japan and North America (24). In low-prevalence areas, the incidence of vertical and horizontal transmission in childhood is low, with most incident infections occurring in adolescence and adulthood through sexual contact, injecting drug use, and other blood-borne exposures (24). Global migration from higher prevalence to lower-prevalence countries is also an important determinant of the burden of chronic HBV in these countries, where the prevalence in migrants generally reflects that of their country of origin (24).

### Epidemiology of GDM

Analysis conducted by the International Diabetes Foundation (IDF) including 51 studies across 41 countries, estimated 20.4 million or 15.8% of live births in 2019 to women aged 20–49 were affected by a form of Hyperglycaemia in Pregnancy (HIP); of which 83.6% were due to GDM (27). The vast majority of cases of HIP (86.8%) are found in low to middle income countries, where access to antenatal care is often limited (27). Regional differences in the prevalence rates of HIP and GDM have been reported. The 2019 IDF analysis reports the highest age-adjusted comparative prevalence rates of HIP in Southeast Asia (27%) followed by North America and Caribbean (20.8%), and lowest in Africa (9.6%) and Middle East and North Africa (7.5%) (27). In contrast, a systematic review conducted between 2005 and 2015 reports the following median GDM prevalence rates for the same regions: Middle East and North Africa (12.9%), Southeast Asia (11.7%) Africa (8.9%), North America and Caribbean (7%) (28). The discrepancy on data estimates of global GDM prevalence over time has been suggested to be partly attributable to a lack of consensus and uniformity in GDM screening standards and diagnostic criteria across regions and studies (28).

Differences in rates of GDM are reported across individuals with different countries of birth. The reasons underlying these differences are still to be further elucidated, but accumulating evidence suggests the mechanisms could be multi-faceted, including differences in body composition, lifestyle (diet and physical activity), cultural practices and genetic susceptibility (28, 29).

### Ethnic and regional influences on GDM incidence among people with chronic HBV

Several studies report an association between chronic HBV and development of GDM in pregnancy (3–7, 11, 12, 18–21) although this has not been a consistent finding (8–10, 13–17). The vast majority of studies exhibiting a positive association between GDM and chronic HBV have been conducted in areas of high-intermediate HBV prevalence, namely Central and South East Asia (3, 4, 6, 7, 11–14, 19–21). In such studies, HBV prevalence ranges between 2.4%–11.3% (3–7, 11, 12, 18–21). The incidence of GDM amongst individuals with chronic HBV in this population ranges from 1.2% (13) to as high as 32.9% (21). Coincidentally, regions of high HBV prevalence also represent ethnic groups with an increased propensity to develop GDM (25, 28) and therefore it is challenging to distinguish whether the increased risk is attributable to socio-demographic factors or chronic HBV status.

Not all studies from this region have found the same association. One large prospective cohort study in Eastern China did not demonstrate a significant difference in the incidence of GDM among people with chronic HBV compared to uninfected individuals (1.2% vs. 1.1% respectively) (13). The prevalence of chronic HBV infection in this population (2.5%) was much lower than that reported by other Chinese studies (4.2–11.3%) (3, 4, 6, 11). Similarly, Sirilert et al. also found no significant relationship between chronic HBV and GDM amongst their ethnic Thai population (14).

Studies from regions of low HBV prevalence but consisting of a multi-ethnic population have yielded HBV prevalence rates between 0.1%–0.5% (5, 9, 10, 16–18) and concurrent GDM incidence of 2.2–14.3% (5, 9, 10, 16–18). The majority of these studies reported no increased risk for GDM amongst those with chronic HBV (9, 10, 16, 17). However, a large population-based US cohort study reported significantly higher GDM incidence amongst individuals with chronic HBV than healthy controls (7.2% vs. 4.4% respectively) (18). It must be noted that in this study, despite representing 1.5% of the sample group, individuals of Asian ethnicity had a higher HBV prevalence rate (932.1 per 100 000 live births), almost 30-fold greater than the rate seen among women of Caucasian background (34.8 per 100 000 live births) (18). The study unfortunately did not comment on GDM incidence per ethnicity (18). Furthermore, a recent Australian study demonstrated an increased incidence of GDM in women with HBV born in low HBV prevalence regions (such as Southern/Eastern Europe), but no significant association between GDM and HBV in women from traditionally higher prevalence regions of birth (5). They reported an overall incidence risk ratio for GDM of 1.2 (95% CI 1.1–1.3) among women with HBV after adjusting for region of birth, BMI, parity, age and smoking status. This line of evidence implicates a risk of GDM unrelated to the ethnic or regional influences previously purported, and hints towards intrinsic viral factors as a possible mechanism.

## Possible mechanisms

### Inflammatory basis

Low grade systemic inflammation appears to plays a role in the pathophysiology of GDM (30–32). Studies have shown an independent association between elevated C-reactive protein (CRP) levels measured early in the second trimester and subsequent GDM (32, 33). Chronic HBV infection, as a chronic inflammatory state, has been proposed to promote a greater predisposition to developing GDM (13, 34, 35). A prospective cohort study measuring serum ALT (alanine transferase) as a marker of hepatic inflammation, across various timeframes in pregnancy found people with chronic HBV had significantly higher ALT levels than controls (13). However, no significant difference in the incidence of GDM between those with chronic HBV and control groups was found (13). Approximately 60%–80% of individuals with chronic HBV have persistently normal ALT levels (36), and therefore this may not be the best marker of inflammation in chronic HBV infection. Tumour necrosis factor alpha (TNF-α) is a cytokine that has been shown to be crucial in facilitating the immune-mediated virological control of chronic HBV, and subsequent collateral hepatocyte damage (37). TNF-α is also known to be synthesised by the placenta and studies have shown significantly higher serum concentrations in pregnant women with GDM compared to pregnant women without GDM (30, 31). To date, there are no studies that have directly explored the relationship between TNF-α and GDM in women with chronic HBV.

Ferritin, is an acute phase reactant, and is another inflammatory marker of interest in elucidating an inflammatory basis to the development of GDM among those with chronic HBV (19, 21). Lao et al. (21) first demonstrated a significantly higher level of ferritin and transferrin concentrations in HBsAg (hepatitis B surface antigen) positive women with GDM. In a subsequent retrospective cohort study they further showed a significantly lower prevalence of iron deficiency anaemia among HBV positive pregnant women, thereby inferring higher iron stores in this group (30). However, no formal data was available on the iron studies of this maternal population for further quantitative analysis.

The interplay between ferritin and GDM may be more complex than merely reflecting inflammation. Chronic viral hepatitis has been associated with iron-overload and high serum hepcidin, a peptide hormone involved in the homeostasis of iron (38). Excess iron can directly affect insulin synthesis/secretion and enhance oxidation of free fatty acids, in turn decreasing glucose utilisation in muscles and promoting gluconeogenesis in the liver, ultimately leading to insulin resistance (39).

### Viral factors

Chronic HBV infection is a dynamic process characterised by a complex interplay between the virus and the immune system's attempts to control viral replication and manage clearance. In addition, during pregnancy, major physiological adaptations occur in the maternal immune system to avoid detrimental responses against the allogeneic fetus (40). These changes have been purported to interfere with the immune modulation of chronic HBV, thereby facilitating HBV replication (35).

Hepatitis B envelope antigen (HBeAg) and HBV DNA viral load serve as markers of viral replication in chronic HBV (14, 35). HBeAg and HBV DNA are often measured in HBsAg positive women in pregnancy to assess the need for antiviral therapy to reduce the risk of MTCT. Lao et al. (*2013*) reported that HBV DNA was detected in 19.6% and 30.4% in the second and third trimesters respectively of HBsAg positive mothers without detectable HBV DNA in the first trimester (41). It has been hypothesised that positive maternal HBeAg status and high HBV viral load could incite a more vigorous immune response exacerbating inflammation thereby worsening insulin resistance, leading to GDM (4, 6, 7, 14, 39). However, with the exception of one study (39), recent literature exploring this proposition has largely shown no association (4, 6, 7, 14).

Sirilert et al. found no significant difference in GDM incidence between their HBsAg positive and negative groups, but noted that HBsAg positive women were more likely to develop GDM if they were also HBeAg positive (14). A single centre study explored the association between viral load status (categorising HBV positive women as HBV DNA positive if levels ≥10^3^ copies/ml and negative if <10^3^ copies/ml) and glycaemic control in pregnancy (6). Fasting blood glucose, 2 h post oral glucose levels, glycated haemoglobin (HbA1c) and incidence of GDM were significantly higher in both of the HBV positive groups in comparison to the control group, however there was no difference between the two viral load statuses (6). Two similarly designed retrospective cohort studies, despite showing significant association between positive HBsAg status and GDM, both found no relationship between positive HBeAg status or distribution of viral load (4, 7).

A more recent study (39) has quantified viral load using IU/ml; categorising low, medium and high loads as: less than 10^3^ IU/ml, 10^3^ -10^6^ IU/ml and greater than 10^6^ IU/ml respectively. This study reported that a high HBV DNA load (>10^6^ IU/ml) was an independent risk factor for GDM among HBsAg positive pregnant women (OR 2.65 [1.39, 5.04], *p* < 0.05) after adjusting for age and pre-pregnancy BMI.

## Association between diabetes and other liver conditions

### Hepatitis C

There is a well established association between chronic hepatitis C virus (HCV) infection and type 2 diabetes mellitus (T2DM), with a T2DM prevalence ranging between 18%–35% among individuals with HCV (42). Hui et al. reported higher levels of fasting insulin, C-peptide and HOMA-IR (a physiological homeostasis model that predicts insulin resistance) amongst HCV infected individuals with no or minimal hepatic fibrosis compared to their healthy controls matched for BMI and waist-to-hip ratio (43). They also showed that increased HOMA-IR values were associated with a higher grade of portal inflammation (43), a hallmark of HCV that correlates with fibrosis progression.

There is emerging evidence to suggest that direct-acting antiviral agents (DAA) have an impact on glycaemic control (44). Patients with diabetes and chronic HCV undergoing DAA therapy, who achieve a sustained virological response, have been shown to have improved blood sugar control post-DAA therapy, prompting a reduction in dose or discontinuation of oral hypoglycaemic agents or insulin therapy (44). The purported mechanisms for HCV-induced insulin resistance include, but are not limited to: direct viral effects on the downregulation of glucose transporters and release of pro-inflammatory cytokines leading to changes to the insulin signalling pathway, as well as promoting hepatic steatosis (42–44). The above mechanisms may also play a role in the development of GDM among HCV-infected pregnant women. A large population-based cohort study conducted in the US demonstrated a greater risk of developing GDM among women with chronic HCV, especially in the context of increased gestational weight gain (aOR 2.5 [1.0–6.0]) (45). Similar findings were confirmed in two further US cohort studies (16, 18). However this association was not demonstrated in a population-based cohort study conducted in Sweden (10).

### Non-viral hepatitides

Given the liver's crucial role in glucose metabolism and homeostasis, there have been studies looking into the relationship between non-infective liver conditions and the development of GDM. Autoimmune hepatitis (AIH) has been noted to be a risk factor for adverse pregnancy outcomes (46), yet studies looking into its relationship with GDM have been inconclusive. A retrospective nationwide cohort study undertaken in Sweden reported GDM in 4.7% of AIH pregnancies compared to 1.1% of control pregnancies, with an adjusted risk ratio of 4.35 (95% CI 2.21–8.57) (46). The study also found significant associations between AIH and many other autoimmune diseases, and thus the authors reasoned this was the basis for the increased rate of GDM. On the contrary, a subsequent multicentre study found no significant association between AIH flares in pregnancy and adverse pregnancy outcomes, including GDM (47).

Non-alcoholic fatty liver disease (NAFLD) known to be the hepatic manifestation of metabolic syndrome (48) has been demonstrated in various studies to increase insulin resistance and thus GDM. In their prospective study, De Souza et al. showed that NAFLD assessed by ultrasound at 11–14 weeks gestation independently predicted poor glycaemic control in pregnancy (determined by a fasting 75 g OGTT at 24–28weeks), after adjusting for maternal age, ethnicity, family history of T2DM, maternal BMI and change in BMI throughout pregnancy (49). Similarly, another study using elevated ALT, in the absence of viral hepatitis and alcohol abuse, as a surrogate marker for NAFLD demonstrated a positive relationship between raised ALT levels in pregnancy and subsequent GDM (48). A recent meta-analysis which included studies looking at the presence of imaging-confirmed NAFLD in the antepartum and subsequent GDM reported an overall prevalence of 26% for GDM amongst the NAFLD cohort with a significant pooled odds ratio of 2.9 (95% CI 1.0–8.4) (50).

## Future direction

Given the implications of GDM in pregnancy, and the association reported with chronic HBV, there is a need to elucidate the causal mechanisms behind this relationship. This may allow for early interventions in pregnancy in women infected with chronic HBV to reduce the development of GDM.

The majority of studies in chronic HBV have been conducted in Asia, a region of high HBV and GDM prevalence. However, studies conducted in low HBV prevalence regions have also exhibited a positive association between chronic HBV and GDM (5, 18). This must be further explored, especially in the context of multi-ethnic populations. Widespread immigration of Asian and African ethnicities (whom traditionally have a lower mean BMI) to countries with increased prevalence of obesity such as the United States or Australia, has led to overweight and obesity rates among these ethnic groups rising dramatically—placing pregnant women in these groups at higher risk of GDM (29).

The predominant hypothesis for the pathogenesis of GDM in pregnant women with chronic HBV is the presence of a chronic inflammatory state (13, 34, 35). Studies to date have explored the associations between markers of inflammation such as CRP (32, 33) and ALT (13) and GDM. Future studies should consider exploring TNF-α as the possible inflammatory basis for the relationship between chronic HBV and GDM. Studies examining the effect of HBeAg and HBV-DNA viral load on the development of GDM have yielded inconsistent results thereby making it difficult to ascertain if viral factors are at play. Thus, more prospective studies are needed to better characterise the viral activity of chronic hepatitis during pregnancy and its impact on GDM development. Moreover, demographic and viral factors such as age at infection, ethnic background, region of birth, viral genotype and phase of infection may potentially impact the degree of replication activity seen in women with chronic HBV during pregnancy (12, 35). Future studies must also take this into account.

Finally, there have been no studies looking into the effects of HBV antiviral therapy on insulin resistance during pregnancy. Currently antiviral therapy for chronic HBV is initiated in the third trimester in the context of high viral loads in order to lower the risk of MTCT. In line with the emerging evidence of DAA therapy and its potential impact in improving glycaemic control in individuals with chronic HCV, future prospective studies looking into the association of HBV antiviral therapy on glycaemic control in pregnancy may be able to shed more light on the direct effects of HBV on GDM.
